# High-Fat-Diet–Induced Oxidative Stress Linked to the Increased Colonization of Lactobacillus sakei in an Obese Population

**DOI:** 10.1128/spectrum.00074-21

**Published:** 2021-06-30

**Authors:** Jee-Yon Lee, Eunsoo Bae, Hwa Young Kim, Kang-Mu Lee, Sang Sun Yoon, Duk-Chul Lee

**Affiliations:** a Department of Family Medicine, Severance Hospital, Yonsei University College of Medicine, Seoul, Republic of Korea; b Chaum Life Center, CHA Bundang Medical Center, School of Medicine, CHA University, Seoul, Republic of Korea; c Department of Microbiology and Immunology, Brain Korea 21 Project for Medical Sciences, Yonsei University College of Medicine, Seoul, Korea; d Institute for Immunology and Immunological Diseases, Yonsei University College of Medicine, Seoul, Korea; Broad Institute

**Keywords:** *Lactobacillus sakei*, obesity, gut microbiome, reactive oxygen stress, inflammation, high-fat diet

## Abstract

Obesity is a major public health problem related to various chronic health conditions. *Lactobacillus* species has been reported in obese individuals; however, its role is unknown. We compared the abundance and composition of *Lactobacillus* species by analyzing feces from 64 healthy control subjects and 88 obese subjects. We isolated one *Lactobacillus* strain from the feces of a subject with obesity and further analyzed its genetic and molecular features. We found that an increased abundance and higher prevalence of Lactobacillus sakei distinguished the fecal microbiota of the obese group from that of healthy subjects and that it was related to the increased levels of reactive oxygen species (ROS) induced by higher fat intake. The *L. sakei* ob4.1 strain, isolated from the feces of a subject with obesity, showed high catalase activity, which was regulated by oxidative stress at the gene transcription level. *L. sakei* ob4.1 maintained colon epithelial cell adhesion ability under ROS stimulation, and treatment with saturated fatty acid increased colon epithelial ROS levels in a dose-dependent manner; however, *L. sakei* ob4.1 did not change the level of fat-induced colon epithelial ROS. Exposing mice to a high-fat diet revealed that high-fat-diet–induced colon ROS was associated with the increased colonization of *L. sakei* ob4.1 through catalase activity. Four-week supplementation with this strain in mice fed a high-fat diet did not change their body weights or ROS levels. A high-fat diet induces changes in the colon environment by increasing ROS levels, which provides a colonization benefit to an *L. sakei* strain with high catalase activity.

**IMPORTANCE** *Lactobacillus* provides many health benefits; its various species are widely used as probiotics. However, an increased abundance of *Lactobacillus* has been reported in obesity, and the role of *Lactobacillus* strains in obesity remains unknown. We found a high abundance of the Lactobacillus sakei species in a group of obese subjects and examined its relationship with a high-fat diet and reactive oxygen species (ROS) in the feces. To find the underlying mechanism, we analyzed and characterized an *L. sakei* strain isolated from a severely obese individual. We found that higher gut oxidative stress could link high-fat-diet–induced obesity and *L. sakei.* This translational research identifies the roles of the host gut environment in the colonization and survival of *L. sakei*.

## INTRODUCTION

Obesity is a multifactorial disease that involves both genetic and environmental factors (1, 2); therefore, identification of its risk and preventive factors is needed to reduce the incidence of obesity and obesity-related complications.

The relationship between gut microbiota and obesity has been widely studied (3, 4). The gut microbiota is known to cause obesity by various mechanisms, including the modulation of host genes to affect the extraction of energy from the diet (5). On the other hand, changes in the gut environment induced by obesity can cause gut dysbiosis. The consumption of a high-fat diet, one of the main causes of obesity, is considered to play a key role in changing the gut environment. A high-fat diet increases the levels of oxidative stress in the colon (6, 7) and affects the composition of the colonic microbiota (8, 9). Hence, a reciprocal relationship exists between obesity and the gut microbiota.

Although the causal relationship is not yet understood, obesity has been associated with specific groups of gut bacteria, including *Lactobacillus*. An increased abundance of commensal *Lactobacillus* (10), particularly Lactobacillus sakei (11), in obese populations has been reported in case-control studies. However, many strains of *Lactobacillus* are used as probiotics, and some strains of *Lactobacillus* have shown beneficial effects on obesity and obesity-related complications (12–14) in clinical trials. There is no evidence that the consumption of *Lactobacillus* as probiotics can induce obesity in a healthy population (15), and the mechanism underlying the relationship between an increase in *Lactobacillus* and obesity is still unknown and likely to vary across strains.

*Lactobacillus* has been reported to show high diversity in its resistance to oxidative stress across species (16, 17) and strains (18), based on its catalase activity. For example, *L. sakei*, one of the *Lactobacillus* strains correlated with obesity, is highly resistant to oxidative stress via catalase activity (19, 20) and has a high degree of intraspecies diversity regarding the response to oxidative stress (18) Therefore, we hypothesized that colonization by different *Lactobacillus* strains could be determined by the oxidative stress levels in the colon, such as those that can be induced in obesity by a high-fat diet. In this study, we compared the abundance and patterns of commensal *Lactobacillus* in obese and healthy subjects and investigated the risk factors that could explain the relationship between *Lactobacillus* species and obesity. Then, we isolated a strain of a *Lactobacillus* species related to obesity and further investigated its genetic and molecular characteristics as they relate to colonic oxidative stress in obesity.

## RESULTS

### Clinical characteristics of healthy control and obese subjects.

In this study, we enrolled 88 adults with obesity and 64 healthy control subjects without obesity. No significant difference was found between the obese and control groups regarding age (*P* = 0.22), sex (*P* = 0.20), or social habits such as exercise (*P* = 0.24) and alcohol consumption (*P* = 0.13). All the participants were nonsmokers. A higher intake of daily calories (*P* = 0.01), calorie-adjusted fat (*P* < 0.01), and calorie-adjusted saturated fat (*P* < 0.01) was found in the obese group (see Table S3 in the supplemental material).

### Lactobacillus sakei abundance was higher in the obese group.

**FIG 1:**
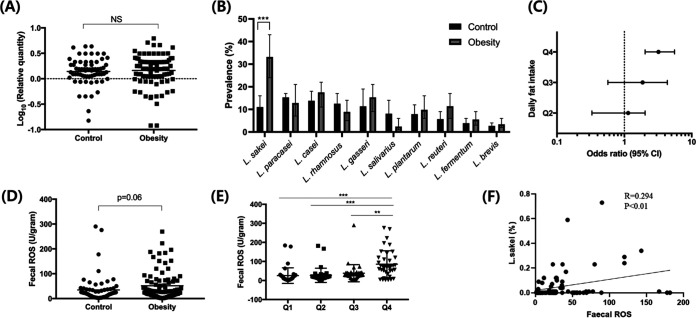
Association between high fat intake in the obese group and increased gut oxidative stress and Lactobacillus sakei colonization. A total of 64 healthy control subjects (BMI between 19 and 23 kg/m^2^) and 88 obese subjects (BMI > 25 kg/m^2^) participated in this study. Fecal samples were collected from each participant, and all participants completed a questionnaire regarding food intake. Participants were categorized into four groups according to the quartiles of fat consumption (1st quartile [Q1], <16.66 g/1,000 kcal/day; 2nd quartile [Q2], 16.66 to 22.02 g/1,000 kcal/day; 3rd quartile [Q3], 22.02 to 42.65 g/1,000 kcal/day; 4th quartile [Q4], >42.65 g/1,000 kcal/day). (A) The relative abundance of the genus *Lactobacillus* in feces was determined via real-time PCR targeting the 16S rRNA gene. (B) The presence of *Lactobacillus* species in feces was determined using species-specific PCR. (C) Odds ratios and 95% CIs for the prevalence of a positive culture of *L. sakei* species were calculated using a multivariate logistic regression analysis adjusted for age, sex, and BMI. A nutritional analysis was performed using CAN-PRO software 5.0. (D, E, and F) The levels of fecal ROS were measured using an ELISA. Each symbol represents data from one individual subject (A, D, and E); each line represents the odds ratio with the 95% confidence interval (B). **, *P < *0.01; ***, *P < *0.001; NS, *P > *0.05. *P* values were calculated using the Mann-Whitney test (A), Fisher’s exact test (B), Student’s *t* test (D), one-way ANOVA followed by Tukey’s multiple-comparison tests (E), or Pearson’s correlation analysis (F).

To compare the abundance and composition of *Lactobacillus* species between the two groups, we quantified the relative abundance of *Lactobacillus* in their feces using real-time PCR, and we found no difference between the two groups (Fig. 1A). Because the high diversity across *Lactobacillus* species could obscure the relationship between obesity and *Lactobacillus*, we next performed a species-specific PCR analysis. The obese group was colonized with *L. sakei* more often than the control group (*P* < 0.01) (Fig. 1B; see also Table S4). The prevalence of other *Lactobacillus* species did not differ significantly between the two groups (Fig. 1B; Table S4).

Next, we selected fecal samples from 80 participants (47 in the healthy control group and 33 in the obesity group) among the total 154 participants and further compared the relative abundances of microbiota species between the two groups by analyzing 16S rRNA gene amplicon sequencing (microbiota profiling). Consistent with a previous report (21), the microbiota composition of the obese group was characterized by an increased relative abundance of the phylum *Firmicutes* and a reduced abundance of the phylum *Bacteroidetes* compared to that in healthy subjects. Notably, an elevated relative abundance of the class *Clostridia* distinguished the fecal microbiota composition of obese subjects from that of the healthy subjects (see Fig. S1A). An elevated abundance of the class *Clostridia* is known to correlate with a high-fat diet in obesity (22). We also compared the relative abundance of fecal microbiota at the species level. Consistent with the results from the species-specific PCR analysis, the microbiota composition from obese subjects was characterized by an increased abundance of the species *L. sakei* compared with the microbiota in healthy subjects (Fig. S1B).

### Relationships among fat consumption, ROS, and *L. sakei* abundance.

Because the obese group consumed more fat than the control group, we analyzed the relationship between fat consumption and *L. sakei* abundance. High fat consumption is one of the principal reasons for obesity, and it affects the composition of the gut microbiota independent of obesity development (23, 24). We categorized the participants into four groups according to the quartiles of fat consumption (1st quartile [Q1], <16.66 g/1,000 kcal/day; 2nd quartile [Q2], 16.66 to 22.02 g/1,000 kcal/day; 3rd quartile [Q3], 22.02 to 42.65 g/1,000 kcal/day; 4th quartile [Q4], >42.65 g/1,000 kcal/day). Notably, participants in the highest fat consumption group were approximately 3.1 times (odds ratio [OR], 3.21; 95% confidence interval [CI], 2.03 to 4.57; *P* < 0.01) more likely to have the *L. sakei* strain in their feces than those in the lowest fat intake group after adjusting for age, sex, and body mass index (BMI) (Fig. 1C). Saturated fatty acids increase reactive oxygen species (ROS) levels in the gut (6, 7), and *L. sakei* contains heme-dependent catalase, despite the fact that most *Lactobacillus* species are catalase negative (19). Therefore, we hypothesized that the catalase activity of *L. sakei* is the mechanism that underlies the relationship between a high-fat diet and *L. sakei*. To investigate that hypothesis, we measured the ROS levels in the feces of each participant. We found a trend of increased ROS levels in the feces from the obese group compared with that from the control group (*P =* 0.06) (Fig. 1D). When we categorized the participants according to their fat intake, the ROS levels in the feces were found to increase significantly with an increase in fat intake after adjusting for BMI (Q1 versus Q4, *P* < 0.001; Q2 versus Q4, *P* < 0.001; Q3 versus Q4, *P* < 0.01) (Fig. 1E). That suggested that the higher levels of ROS might arise from fat intake rather than from obesity itself. To investigate the association between fecal ROS and L. *sakei*, we performed a Pearson correlation analysis between the fecal ROS levels and relative abundance of L. *sakei* in 80 participants. We found a significant positive relationship (*r* = 0.293, *P* < 0.01) between the relative abundance of *L. sakei* and fecal ROS levels (Fig. 1F). Although the cross-sectional analysis precluded the determination of a causal relationship, this result suggests that the higher abundance of *L. sakei* found in obese subjects might be associated with the increased colonic ROS levels induced by a high-fat diet.

### A Lactobacillus sakei strain was isolated from the feces of obese subjects.

To further investigate the characteristics of *L. sakei* that might be related to high-fat-diet–induced ROS, we isolated an *L. sakei* strain from the feces of obese subjects. Fresh feces from 15 subjects in the obesity group were used for this purpose. *Lactobacillus*-specific culture to confirm the presence of the *L. sakei*-specific *katA* gene and 16S rRNA gene sequencing were used for isolation. Only one strain of *L. sakei* was isolated from one subject, and we named it *L. sakei* ob4.1. Severe obesity (BMI 32.15 kg/m^2^) and high fat consumption (37.28 g/1,000 kcal/day) were found in the host of *L. sakei* ob4.1 (see Table S5).

### *L. sakei* ob4.1 showed higher resistance to oxidative stress than *L. sakei* DSM 20017.

**FIG 2:**
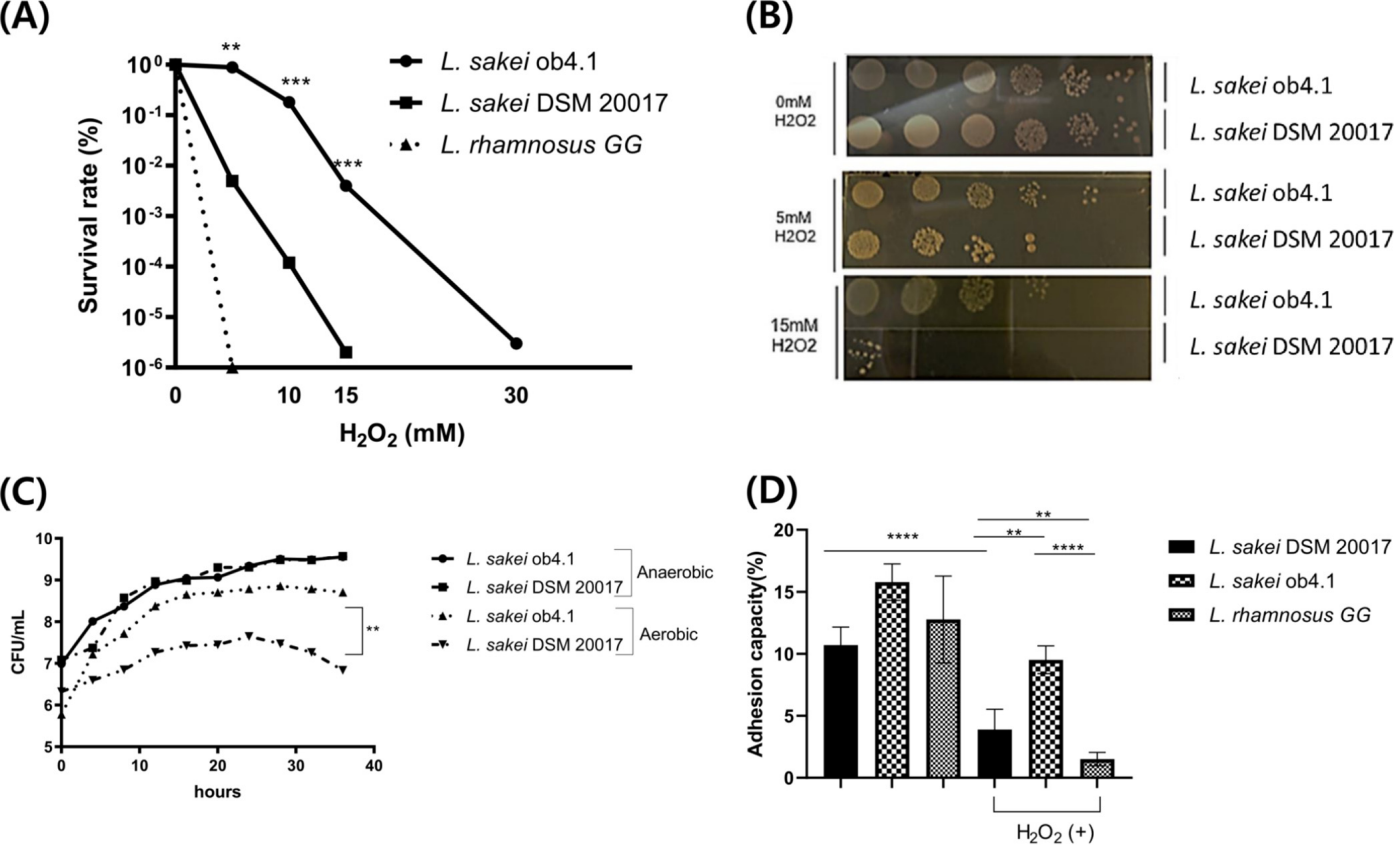
Lactobacillus sakei ob4.1 was more resistant to H_2_O_2_ than *L. sakei* DSM 20017. *L. sakei* ob4.1 was isolated from one participant from the obese group. For comparison, *L. sakei* DSM 20017 (purchased from KCTC) was used as the reference strain, and L. rhamnosus GG (ATCC 53103) (purchased from ATCC) was used as the negative control. (A) The survival rate was calculated by counting the number of viable cells of each *L. sakei* strain in MRS medium after 1 h in 0, 5, 10, 15, and 30 mM H_2_O_2_. (B) Serial dilutions of bacterial cells were inoculated onto MRS plates. The short-term survival ratio of *L. sakei* ob4.1 was significantly higher than that of *L. sakei* DSM 20017 in the presence of each concentration of H_2_O_2_ (5 mM, *P* < 0.01; 10 mM, *P* < 0.001; 15 mM, *P* < 0.001). (C) Overnight-grown cultures of *L. sakei* ob4.1 or DSM 20017 were diluted in MRS medium (1:1,000) and incubated at 37°C, either aerobically using a shaking incubator (200 rpm) or anaerobically in an anaerobic jar with GasPak (BD). CFU were determined by measuring the optical density at 600 nm every 4 h for 3 days. (D) A bacterial suspension of approximately 10^6^ CFU/ml was added to a monolayer of Caco-2 cells with or without 0.5% H_2_O_2_, and then the cells were incubated at 37°C for 1 h. Nonadherent bacteria were removed, the number of viable bacterial cells was determined via the spread plate method on MRS medium, and the cells were then incubated at 37°C for 48 h. Adhesion ability was calculated as the percentage of adhered cells with respect to the total number of bacteria. Bars represent the geometric mean ± standard deviation. **, *P < *0.01; ***, *P < *0.001; ****, *P* < 0.0001. *P* values were calculated using the Student’s *t* test (A and C) or one-way ANOVA followed by Tukey’s multiple-comparison tests (D).

Because our data suggest that high fat intake increases ROS levels in the feces, we explored the resistance of *L. sakei* ob4.1 against oxidative stress. *L. sakei* shows high diversity in its catalase activity across strains (18). For comparison, we chose *L. sakei* DSM 20017 (isolated from rice wine) as a reference strain. For the negative control, we used L. rhamnosus GG (ATCC 53103), which contains no catalase gene (25). The survival of each strain was assessed under oxidative stress conditions generated by H_2_O_2_ or aeration. The short-term survival ratio of *L. sakei* ob4.1 was significantly higher than that of *L. sakei* DSM 20017 in the presence of each concentration of H_2_O_2_ tested (5 mM, *P* < 0.01; 10 mM, *P* < 0.001; 15 mM, *P* < 0.001) (Fig. 2A and B). The long-term survival of the *Lactobacillus* strains under aerobic conditions was evaluated next. After 24 h of aerobic growth, the survival of *L. sakei* ob4.1 was approximately 100 times higher than that of *L. sakei* DSM 20017 (*P* < 0.01) (Fig. 2C). Next, we measured the adhesion ability of the *L. sakei* strains with and without oxidative stress. Compared with L. rhamnosus GG, which has great adhesion ability (26), both *L. sakei* strains showed similar adhesion properties without H_2_O_2_. However, after H_2_O_2_ treatment, *L. sakei* ob4.1 was the only strain that maintained its adhesion ability, showing significantly higher adhesion than *L. sakei* DSM 20017 (*P* < 0.01) (Fig. 2D).

### *L. sakei* ob4.1 had higher catalase expression and activity under oxidative stress.

**FIG 3:**
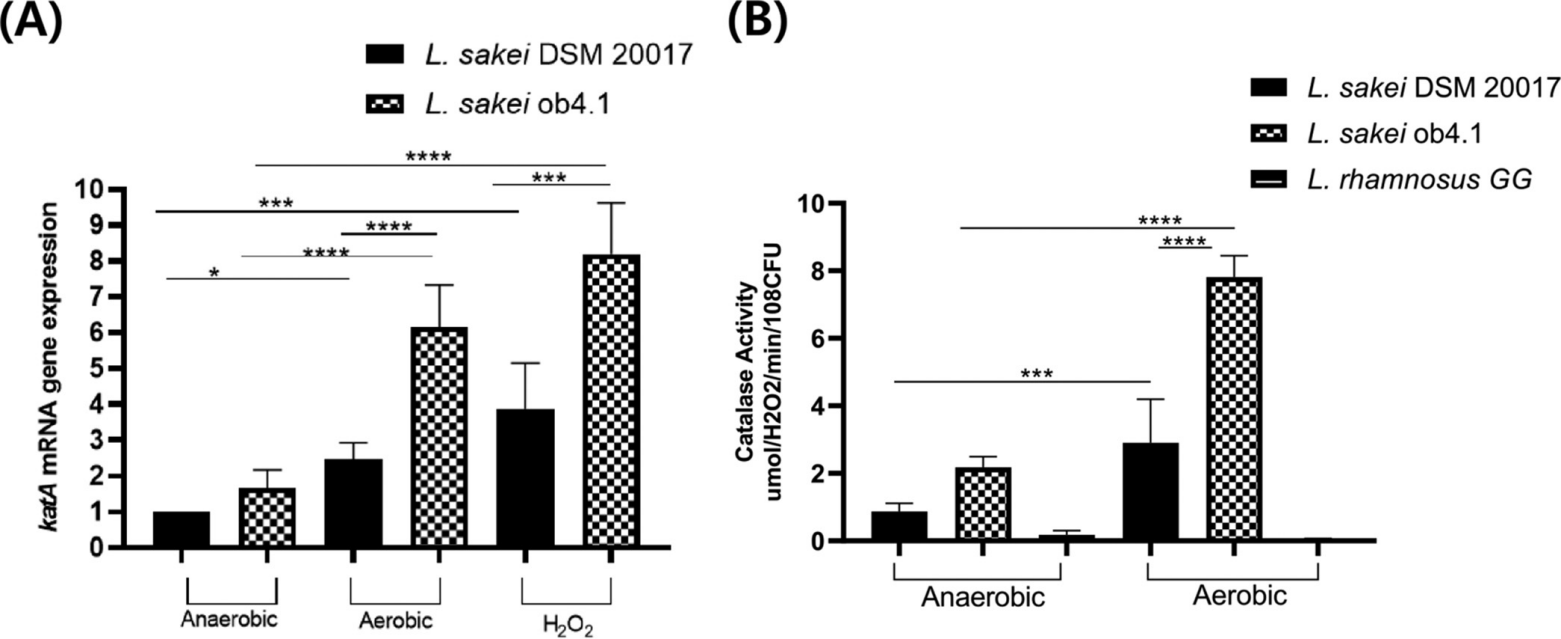
Oxidative stress increased *katA* gene expression and catalase activity in Lactobacillus sakei ob4.1. *L. sakei* ob4.1 and DSM 20017 were cultivated in MRS medium with 30 μM hematin aerobically using a shaking incubator (200 rpm), anaerobically in an anaerobic jar with GasPak (BD), or with 0.2 mM/liter of H_2_O_2_, for 24 h at 37°C; exponential-phase (OD_600_ of 0.5) cells were used for analysis. (A) Transcript levels of *katA* were determined via quantitative real-time PCR using RNA isolated from the indicated *Lactobacillus* strains. (B) Approximately 10^8^ CFU/ml of cells were mixed with 0.8 mM H_2_O_2_ and dichromate in acetic acid followed by measurement of the absorbance at 570 nm. Catalase activity is expressed as micromolar H_2_O_2_ degraded per minute per 10^8^ CFU. Bars represent the geometric mean ± standard deviation. *, *P < *0.05; ***, *P < *0.001; ****, *P < *0.0001. *P* values were calculated by one-way ANOVA followed by Tukey’s multiple-comparison tests.

To understand the genetic basis of the higher resistance of *L. sakei* ob4.1 to oxidative stress, we sequenced its genome and compared the genes involved in oxidative stress with the genome of *L. sakei* DSM 20017. However, we found no difference in the presence or absence of genes involved in oxidative stress between the two *L. sakei* strains. Each *L. sakei* strain contained one catalase gene (*katA*), and 98.5% identity was found across the amino acid sequences of the catalase (see Fig. S2a). Three-dimensional (3D) structures of catalase proteins were predicted, and two-protein structure alignment was performed and compared by using iPBA web server (27). The alignment has a low root mean square deviation of 0.62 Å and 99.6% structural overlap was found across the alignment of two catalase genes. (Fig. 3).

We could not identify genetic factors that explained the higher resistance of *L. sakei* ob4.1 to oxidative stress. However, considering that catalase activity is regulated by oxidative stress at the gene transcription and protein synthesis levels in various *Lactobacillus* species (19, 28), we investigated the effect of oxidative stress on the *katA* mRNA level and catalase enzymatic activity in the *L. sakei* strains. Increased *katA* mRNA expression was detected in both strains when they were grown under aerobic conditions compared to that when grown under anaerobic conditions and in samples treated with H_2_O_2_, but the increase was higher in *L. sakei* ob4.1 (*L. sakei* ob4.1 versus DSM 20017 under aerobic conditions, *P* < 0.0001; under H_2_O_2_, *P* < 0.01) (Fig. 3A). Next, we measured the catalase activities of the *Lactobacillus* strains. A greater increase in catalase activity was found in *L. sakei* ob4.1 grown under aerobic conditions (*L. sakei* ob4.1 versus DSM 20017 under aerobic conditions, *P* < 0.0001) (Fig. 3B). These results collectively suggest that the resistance of *L. sakei* ob4.1 to oxidative stress could be regulated by the extent of oxidative stress at the transcriptional level rather than at the genetic level.

### Palmitate increased colon epithelial ROS levels in a dose-dependent manner, and L. *sakei* did not change the ROS levels.

**FIG 4:**
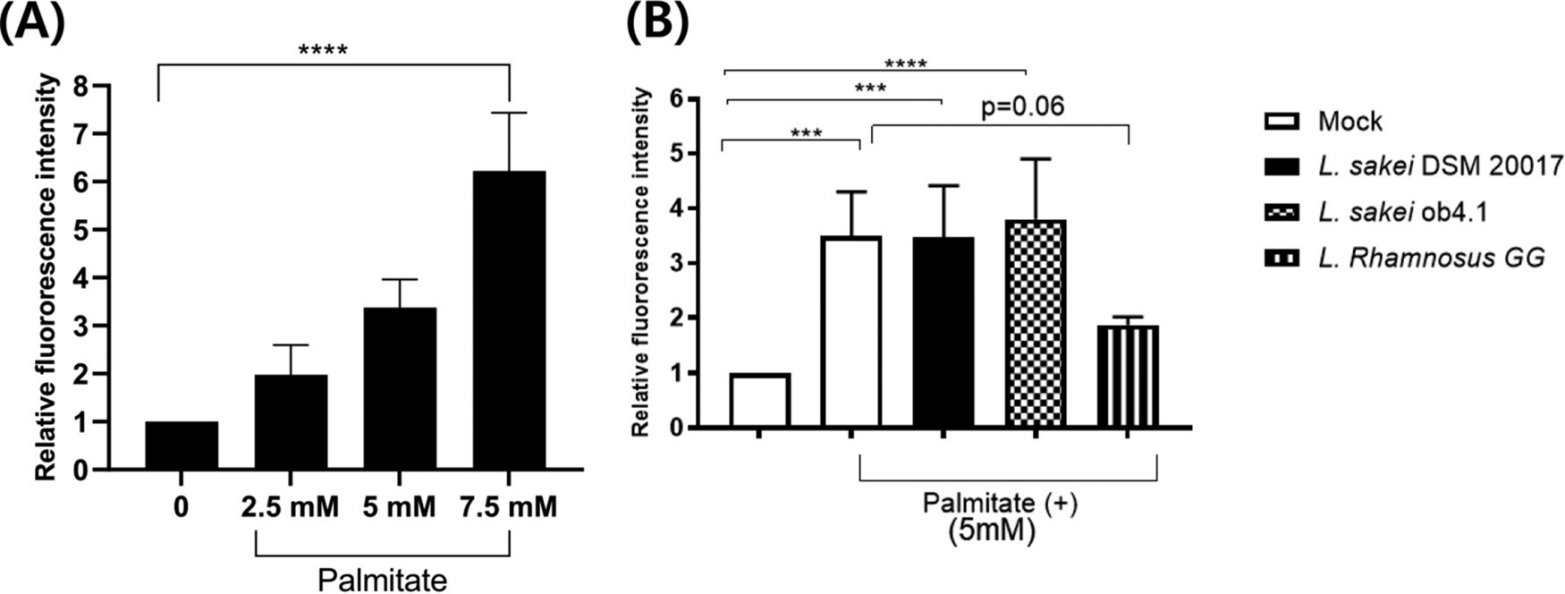
Lactobacillus sakei ob4.1 did not decrease saturated fat-induced ROS in Caco-2 cells. Differentiated Caco-2 cells were treated with different doses of palmitate (0, 2.5, 5, and 7.5 mM) or fatty acid-free bovine serum albumin (mock) and incubated at 37°C and 5% CO_2_ for 6 h. Suspensions of *Lactobacillus* strains containing 10^8^ CFU/ml were added and incubated for another 4 h. ROS production was determined by measuring the ROS-induced fluorescence intensity. Bars represent the geometric mean ± standard deviation. ***, *P < *0.001; ****, *P < *0.0001. *P* values were calculated using a one-way ANOVA trend analysis (A) or one-way ANOVA followed by Tukey’s multiple-comparison tests (B).

Next, we stimulated Caco-2 cells with a saturated fatty acid (palmitate) to identify the effect of fat on host colon epithelium. Palmitate stimulation increased the ROS levels in the colon epithelium in a dose-dependent manner (*P* for trend, <0.0001) (Fig. 4A). However, the high ROS levels were maintained upon treatment with *L. sakei* (mock versus *L. sakei* DSM 20017, *P* = 0.58; mock versus *L. sakei* ob4.1, *P* = 0.79; mock versus L. rhamnosus GG, *P* = 0.06) (Fig. 4B).

### *L. sakei* ob4.1 showed higher colonization in high-fat-diet–induced obese mice than *L. sakei* DSM 20017.

**FIG 5:**
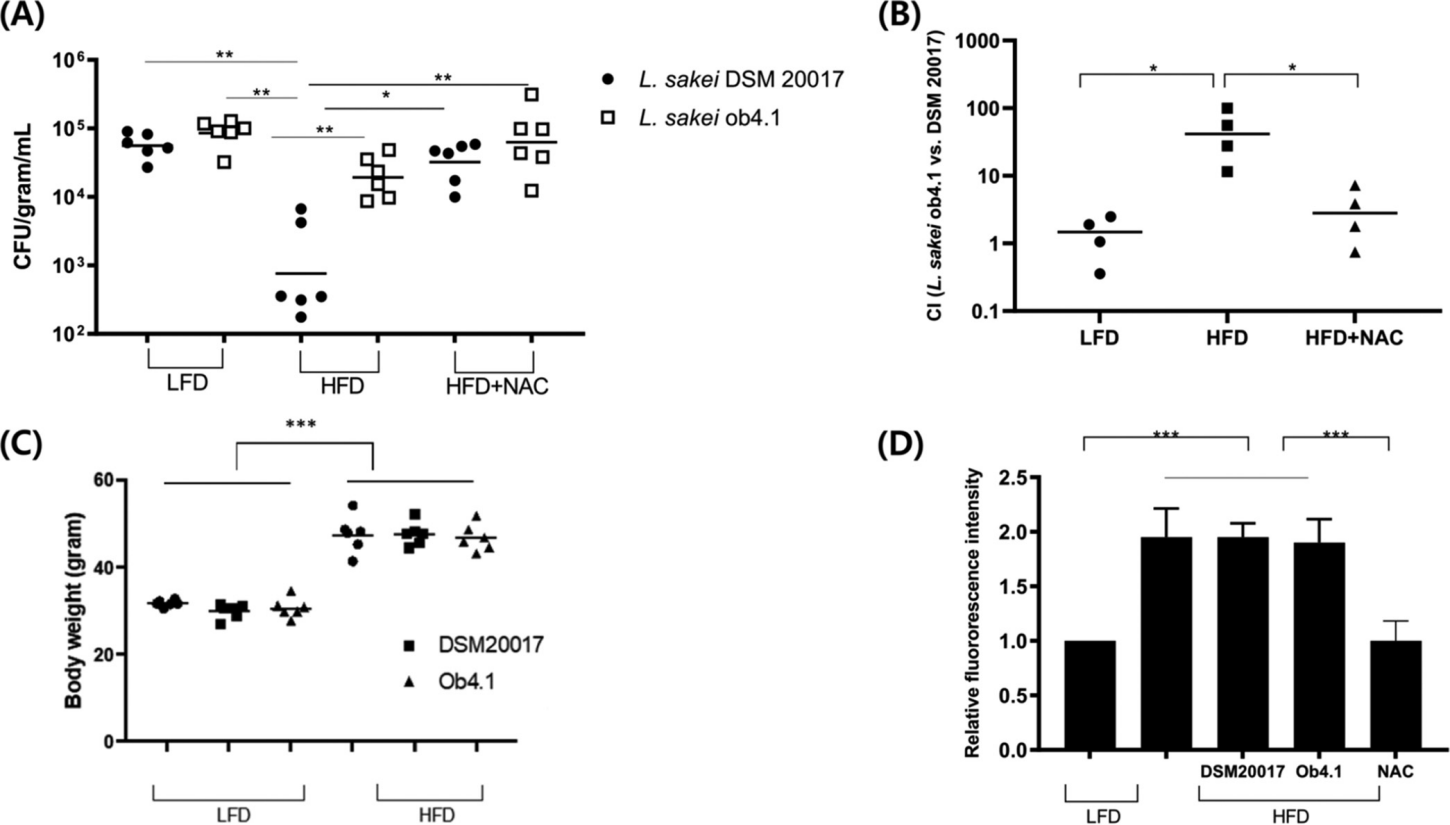
Lactobacillus sakei ob4.1 colonized more than Lactobacillus sakei DSM 20017 in mice fed a high-fat diet. Groups of male mice (*N *= 6) were reared on a high-fat diet (HFD; 60% fat) or a low-fat diet (LFD; 10% fat) for the whole experiment. (A) To investigate the role of a ROS scavenger, a group of male mice (*N *= 6) was given 100 mg/liter of *N*-acetylcysteine (NAC) in their drinking water for the whole experiment. (A) Eight weeks after starting the diet, the mice were mock treated or inoculated with the indicated *L. sakei* strain (1 × 10^9^ CFU/mouse), and the number of *L. sakei* in the colon contents was determined 2 days after inoculation. (B) A pair of mice was cohoused and reared on either a high-fat or low-fat diet. Eight weeks after starting the diet, the mice were separated and inoculated with different *L. sakei* strains (ob4.1 or DSM-20017). The number of bacteria in the colon contents was determined, and a competitive index was calculated using the ratio of bacteria of each strain. (C and D) Four weeks after starting either diet, the mice were mock treated or inoculated with the indicated *L. sakei* strain (1 × 10^9^ CFU/mouse/day) for 4 weeks. (C) Mouse body weight was determined during necropsy. (D) ROS production was evaluated by measuring ROS-induced fluorescence intensity in mouse colon tissue. Dots represent data from individual animals (A, B, and C), and bars represent the geometric mean ± standard deviation (D). *, *P < *0.05; **, *P < *0.01; ***, *P < *0.001. *P* values were calculated using one-way ANOVA followed by Tukey’s multiple-comparison tests.

To investigate the interaction between the *L. sakei* ob4.1 strain and host colon *in vivo*, mice maintained on a high-fat (60%) or low-fat (10%) diet for 8 weeks were inoculated independently with 1 × 10^9^ CFU/mouse of spontaneous rifampin-resistant *L. sakei* strains. After 2 days of gavage, the bacterial numbers in the colon contents were counted. The total number of *Lactobacillus* did not differ between the strains in the mice maintained on a low-fat diet (*P* = 0.17). However, bacterial colonization increased more than 10-fold in *L. sakei* ob4.1-inoculated mice on a high-fat diet compared with that in *L. sakei* DSM 20017-inoculated mice on a high-fat diet (*P* < 0.01). (Fig. 5A and B). This result followed the higher attachment ability of *L. sakei* ob4.1 in the H_2_O_2_-treated Caco-2 cells (Fig. 2D). Next, to confirm that the high-fat-diet–induced ROS caused the higher colonization of L. *sakei* ob4.1 than of *L. sakei* DSM 20017, we added *N*-acetylcysteine (NAC), an antioxidant that scavenges colon epithelial ROS (29, 30), to the drinking water. NAC treatment significantly reduced high-fat-diet–induced colon epithelial ROS levels and eliminated the difference in bacterial colonization between the L. *sakei* ob4.1-inoculated and L. *sakei* DSM 20017-inoculated mice (*P* = 0.48) (Fig. 5A and B). Finally, we wanted to determine the causal relationship between obesity and the colonization of L. *sakei*. The mice on the high-fat diet exhibited higher body weight (*P* < 0.001) (Fig. 5C) and higher colon mucosal ROS levels (*P* < 0.001) (Fig. 5D) than the mice on the low-fat diet. Four weeks of treatment with *L. sakei* ob4.1 or *L. sakei* DSM 20017 did not change the body weights or colon mucosal ROS levels of mice fed a low-fat diet or a high-fat diet (Fig. 5C and D). Our results suggest that increased colonization of Lactobacillus sakei is not a cause of obesity.

## DISCUSSION

*L. sakei* is present in raw meat and is widely used as a starter for the fermentation of sausage (31–33). Recently, *L. sakei* has been found in human feces (34, 35), which could be related to the human diet, such as the consumption of meat. In addition, a higher abundance of L. *sakei* has been reported in the obese population than in the nonobese population (11), although the underlying mechanism for the difference remains unknown. In this study, we found a higher abundance of *L. sakei* in the obese group by using two different methods: species-specific PCR and 16S rRNA gene amplicon sequencing. Our results are consistent with previous findings. Furthermore, we found high-fat-diet–induced ROS to be a potential factor linking obesity and gut microbiota. A high-fat diet increases the levels of colon epithelial ROS (6, 7) and induces colon microbial dysbiosis (8, 9). We also found a significant relationship between the relative abundance of *L. sakei* and fecal ROS levels. Our results provide experimental support for the idea that high-fat-diet–induced colon ROS is responsible for an increased abundance of L. *sakei* in obesity.

Although most *Lactobacillus* strains are sensitive to oxidative stress caused by H_2_O_2_, *L. sakei* is highly resistant to it via catalase activity (19, 20), though there is a high degree of intraspecies diversity in the response to oxidative stress (18). We isolated an *L. sakei* strain (*L. sakei* ob4.1) from the feces of a person with severe obesity; the strain showed higher resistance to oxidative stress than that shown by a reference strain, *L. sakei* DSM 20017, isolated from rice wine.

Oxidative stress occurs because of an imbalance between ROS and the defense system responsible for ROS elimination. A long-term high-fat diet induces oxidative stress in the colon in addition to inducing bacterial dysbiosis by changing the redox status (8). Normally, the colon lumen is hypoxic, and obligate anaerobes are abundant in the healthy gut. However, bacteria with higher resistance to oxidative stress (e.g., Escherichia coli) could have a higher chance of survival in an environment with higher than normal oxidative stress (9). In our study, *L. sakei* ob4.1 had higher resistance to oxidative stress and showed a greater survival rate than *L. sakei* DSM 20017 *in vitro* and higher colonization in the colons of mice fed a high-fat diet. These results collectively suggest that the higher catalase activity of *L. sakei* ob4.1 improves its survival in the colon under high oxidative stress. We could not find a precise molecular explanation for the higher catalase activity of *L. sakei* ob4.1; however, the increased gene transcription and catalase activity under oxidative stress suggests the possibility that it is regulated at the transcriptional and translational levels by the level of oxidative stress.

In our study, 4 weeks of treatment with *L. sakei* ob4.1 did not increase the body weight or colon ROS levels of mice fed either a low-fat diet or a high-fat diet. The risk that ingesting *Lactobacillus* strains as probiotics could promote obesity has been suggested (36). However, insufficient evidence supports that relationship, and the suggestion remains controversial (15, 37). Although we cannot generalize the results from a single strain of *Lactobacillus* isolated from one obese person, our results do not support the hypothesis that *Lactobacillus* strains can cause obesity.

The current study had certain limitations. We did not find the cause of the cross-sectional relationship between the *Lactobacillus* strains and the obese group. We did not analyze consumption of fermented foods which can affect the gut colonization by *Lactobacillus* strains. We compared *L. sakei* ob4.1 with a reference strain isolated from food rather than with a strain isolated from the control group. We did not analyze changes in the microbial community in response to a high-fat diet in mice. Therefore, we could not evaluate the effect of high-fat-diet–induced ROS on the relative abundance of *L. sakei* ob4.1. Additionally, we isolated a single *L. sakei* strain from one obese participant, which does not allow for generalization of our data to the entire obese population.

### Conclusion.

A higher abundance of *L. sakei* correlated with high-fat-diet–induced fecal ROS in an obese population. The higher catalase activity of the *L. sakei* ob4.1 strain, which we isolated from an obese subject, enables the strain to survive better than other strains in the colon under high oxidative stress. Thus, a high-fat diet induces changes in the colon environment by increasing ROS levels, thereby providing a colonization benefit for *L. sakei* strains with higher catalase activity. This translational research identifies the roles of the gut environment induced by host obesity in the colonization and survival of *L. sakei*.

## MATERIALS AND METHODS

### Study participants.

All subjects participated in the study voluntarily, and written informed consent was obtained from each subject. This study complied with the Declaration of Helsinki and was approved by the Institutional Review Board of Yonsei University College of Medicine and CHA Bundang Medical Centre. Individuals who visited the hospital (Department of Family Medicine of Severance Hospital and Chaum Hospital) for either a regular health check-up or to reduce their body weight were recruited as participants. Obese subjects were those whose body mass index (BMI) was >25 kg/m^2^, and control subjects were healthy participants older than 19 years with a BMI between 19 and 23 kg/m^2^. We excluded individuals with a history of any type of cancer, inflammatory bowel disease, or abdominal surgery. Subjects who took probiotics or antibiotics within 4 weeks of study participation were also excluded. Overall, 64 healthy control subjects and 88 obese subjects were included.

### Questionnaire.

All participants completed a questionnaire about their lifestyles, such as their physical activity, smoking, and alcohol consumption. Regular exercise was defined as activity of more than a moderate degree (walking more than 5 times/week for 30 min, moderate-intensity physical activity more than 5 times/week for 30 min, or vigorous-intensity physical activity more than 3 times/week for 20 min) in the preceding week. Smoking was defined as being a current smoker, and alcohol drinking was defined as drinking ≥70 g/day of alcoholic beverages more than once a week. A 24-h dietary recall was conducted with each participant as a nutrition assessment. The standard form included information about each food item consumed (amount, ingredients, and number of times eaten within the previous 24 h). The nutritional analysis, including total calorie intake, total fat intake, and fatty acid intake, was performed using CAN-PRO software 5.0 (Korean National Society, Seoul, Republic of Korea). Each value was adjusted by calculating the nutrient density and expressing it as total amount/1,000 kcal.

### Stool sample collection.

Participants were given plain sterile tubes with no chemical additives for stool collection. Fresh stool samples (approximately 2 g) were collected and transported to the laboratory within 1 h; 100 mg of fresh stool was used for the *Lactobacillus* culture study, and the rest was stored at −80°C for subsequent analyses.

### Human fecal ROS measurement.

Approximately 100 mg of feces was homogenized in phosphate-buffered saline (PBS) and centrifuged at 3,000 rpm for 20 min at 4°C. The supernatant was used for analysis. The human fecal ROS level was measured using a commercial enzyme-linked immunosorbent assay (ELISA) kit (human ROS ELISA kit; MyBioSource Inc., CA, USA) according to the manufacturer’s instructions.

### 16S rRNA quantitative PCR analysis of total *Lactobacillus*.

Genomic DNA was extracted from the feces using a QIAamp DNA stool minikit (Qiagen, CA, USA) according to the manufacturer’s protocol. The relative abundance of *Lactobacillus* was analyzed via quantitative real-time PCR according to the method described by Yoon et al. (38). Briefly, 2 μl of 10-fold-diluted genomic DNA extracted from the feces was taken as the template, and SYBR green PCR master mix (Applied Biosystems, CA, USA) and appropriate primer sets designed to amplify the 16S region (39) (see Table S1 in the supplemental material) were used to perform quantitative real-time PCR. The transcript levels of the target genes were normalized to the gene expression levels of the housekeeping gene *gapdh*.

### PCR analysis of *Lactobacillus* species.

Genomic DNA was extracted from the feces samples using a QIAamp DNA stool minikit (Qiagen, CA, USA) according to the manufacturer’s protocol. *Lactobacillus* species-specific PCR was performed using primers (Table S1) (40–43) targeted on the 16S-23S rRNA intergenic spacer region. The reaction mixture comprised 100 ng of bacterial DNA from the feces and PCR Supermix high fidelity (Invitrogen, CA, USA). Amplified PCR products were detected via agarose gel electrophoresis using a 1.5% agarose gel with ethidium bromide staining and UV transillumination. The size of the PCR products was compared with that of *Lactobacillus* reference strains (Table S2).

### Isolation of *L. sakei* strains from feces.

To isolate *L. sakei* strains from the feces, 100 mg of fresh stool was suspended in 900 μl of PBS and homogenized. The homogenized sample solutions were serially diluted (10^−2^ to 10^−6^); 50 μl of each dilution was plated in duplicates on de Man-Rogosa-Sharpe (MRS) medium (BD Difco, MA, USA) and incubated anaerobically at 37°C for 48 to 72 h. To exclude non-lactic acid-producing bacteria, each colony with a different morphology was isolated, plated on MRS medium with 1% CaCO_3_, and incubated anaerobically at 37°C for 48 h. Bacteria that produced clear zones around the colonies were selected. The presence of the *L. sakei*-specific *katA* gene was determined via PCR amplification, followed by the isolation of *L. sakei* using a previously described method (20). Sanger sequencing of the 16S rRNA gene was performed to confirm *L. sakei*. Genomic DNA was isolated from each colony using a G-Spin genomic DNA extraction kit (iNtRON Biotechnology Inc., Seoul, Republic of Korea) according to the manufacturer’s instructions. The isolated genomic DNA was used in a PCR with primers 27F and 1492R to amplify the 16S rRNA gene. After purification using a QIAquick PCR purification kit (Qiagen, CA, USA), the PCR products were sequenced by Macrogen Inc. (Seoul, Republic of Korea). The nucleotide sequences were analyzed for sequence similarity using BLAST (http://www.ncbi.nlm.nih.gov/blast), and sequences with a ≥98% match with those in the database were considered to be from the same species. The results were further confirmed using the Ribosomal Data Project taxonomy classifier (44). Those results showed that only one strain was obtained from the feces of obese participants.

### Bacterial community analysis.

Genomic DNA extracted from 400 mg of human stool samples was added to 15 ml of DNA extraction lysis buffer (4% SDS, 50 mM Tris-HCL, 50 mM EDTA, 5,000 mM NaCl) and vigorously homogenized by vortexing for 1 min. Then, 1.4 ml of the homogenized fecal suspensions was transferred to a 2-ml Eppendorf tube, bead beaten for 50 s, and centrifuged at 14,000 g for 10 min. Two hundred microliters of supernatants was transferred to 96-well plates, and 1 μl of supernatant was dissolved in 29 μl of nuclease-free water. The plates were kept at −25°C until they were used in PCR. The extracted DNA was amplified using the 341F and 805R primers targeting the V3 to V4 region of the bacterial 16S rRNA gene (Table S1). The PCR was performed under the following conditions: initial denaturation at 95°C for 3 min followed by 25 cycles of denaturation at 95°C for 30 s, primer annealing at 55°C for 30 s, and extension at 72°C for 30 s, with a final elongation at 72°C for 5 min. Then, secondary amplification was performed to attach the Illumina Nextera barcode with an i5 forward primer (5′-AATGATACGGCGACCACCGAGATCTACAC-XXXXXXXX-TCGTCGGCAGCGTC-3′; X indicates the barcode region) and i7 reverse primer (5′-CAAGCAGAAGACGGCATACGAGAT-XXXXXXXX-AGTCTCGTGGGCTCGG-3′). The condition of secondary amplification was the same as for the primary amplification, except the amplification cycle was set to 8 cycles. The PCR product was confirmed using 2% agarose gel electrophoresis, and the amplified products were purified using a QIAquick PCR purification kit (Qiagen, CA, USA). Equal concentrations of the purified products were pooled together, and short fragments (nontarget products) were removed with an AMPure bead kit (Agencourt Bioscience, MA, USA). The product size and quality were assessed using a Bioanalyzer 2100 (Agilent, Palo Alto, CA, USA) with a DNA 7500 chip. Mixed amplicons from different samples were pooled and subjected to pyrosequencing. The process of sequencing was performed by ChunLab, Inc. (Seoul, Republic of Korea) using an Illumina MiSeq Sequencing system (Illumina, USA) according to the manufacturer’s instructions. During the pyrosequencing data analysis, reads obtained from the different samples were sorted using the unique barcodes of each PCR product. Then, the barcode, linker, and primer sequences were removed from the original read of sequences. Among the sequencing reads, those containing two or more ambiguous nucleotides, those with low quality scores (average, <25), and those shorter than 300 bp were discarded. The Bellerophon method that compares BLASTN search results between the forward half and reverse half sequences was used to detect potential chimeric sequences (45). After removing the chimeric sequences, each read was assigned to a taxonomic classification in the EzTaxon-e database (http://eztaxon-e.ezbiocloud.net) (46). This database contains the 16S rRNA gene sequence of type strains with valid published names and representative species-level phylotypes from either cultured or uncultured entries in the GenBank database, with complete hierarchical taxonomic classification from the phylum to the species. To compare the abundance of taxa among the different groups, the linear discriminant analysis (LDA) effect size (LEfSe) algorithm was used with the Galaxy online interface (http://huttenhower.sph.harvard.edu/lefse/). The LEfSe analysis was performed using the Kruskal-Wallis sum rank test with an alpha value of <0.5, followed by the Wilcoxon rank sum test with an alpha score of <0.05 and a one-against-all strategy for multiclass analysis. An effect size of >2 (on a log scale) was considered significant in this study.

### Whole-genome sequencing.

For genome sequencing, genomic DNA was extracted from *L. sakei* ob4.1 grown in MRS medium using a G-Spin genomic DNA extraction Kit (iNtRON Biotechnology Inc., Seoul, Republic of Korea) according to the manufacturer’s instructions. Thereafter, 20-kb sequencing libraries were prepared using a PacBio DNA template prep kit 1.0 according to the manufacturer’s instructions. The libraries were sequenced using PacBio P6C4 chemistry in an 8-well SMRT Cell v3 in PacBio RSII. The sequenced data were assembled with PacBio SMRT analysis 2.3.0 using the HGAP2 protocol (Pacific Biosciences, USA). Contig construction of the library and whole-genome sequences was performed by ChunLab Inc. (Seoul, Republic of Korea). The process of finding genes and functionally annotating whole-genome assemblies was performed using the EzBioCloud genome database. Protein coding sequences (CDSs) were predicted by Prodigal 2.6.2 (47), and tRNAs were searched using tRNAscan-SE 1.3.1 (48). The search for rRNA and other noncoding RNA was performed using a covariance model and the Rfam 12.0 database (49). The CDSs were categorized into different groups based on their functions with reference to orthologous groups (eggNOG 4.5; http://eggnogdb.embl.de) (50). Next, genome sequences of the *L. sakei* DSM 20017 strain were obtained from the EzBioCloud database (51). Genes involved in oxidative stress responses were searched against the whole genes in *L. sakei* ob4.1 and *L. sakei* DSM 20017 with the key phrase “oxidative stress” using the ChunLab’s comparative genomics tool (http://www.ezbiocloud.net/contents/cg). Pairwise amino acid sequence alignments of *katA* genes between *L. sakei* ob4.1 and DSM 20017 were performed using the BLASTp algorithm.

### Survival rate under the hydrogen peroxide challenge test.

Exponential-phase bacterial cells were centrifuged (6,000 × *g*, 15 min), resuspended in MRS medium containing 0, 5, 10, 15, and 30 mM H_2_O_2_, and incubated at 37°C. After 1 h, H_2_O_2_ was eliminated using bovine liver catalase (10 U/ml; Sigma-Aldrich, MO, USA), and viable cells were counted by plating dilutions on MRS medium.

### Growth conditions of the *L. sakei* strains.

Overnight-grown cultures of *L. sakei* ob4.1 or *L. sakei* DSM 20017 were diluted in MRS medium (1:1,000) and incubated at 37°C either aerobically using a shaking incubator (200 rpm) or anaerobically in an anaerobic jar with GasPak (BD Difco, MA, USA). CFUs were determined by measuring the optical density at 600 nm every 4 h for 3 days.

### Detection of catalase activity in *Lactobacillus* strains.

Catalase activity was analyzed as described previously (52). Briefly, *Lactobacillus* strains were incubated in MRS medium with 30 μM hematin (Sigma-Aldrich, MO, USA), either aerobically using a shaking incubator (200 rpm) or anaerobically in an anaerobic jar with GasPak (BD Difco, MA, USA). The exponential growth-phase bacterial cells (optical density at 600 nm [OD_600_] of 0.5) were harvested and resuspended in PBS at 10^8^ CFU/ml. The resuspended cells were mixed with 0.8 mM H_2_O_2_. An aliquot was mixed with dichromate in acetic acid, and the samples were boiled and centrifuged to remove the cells. The absorbance was measured at 570 nm. Catalase activity is expressed in terms of micromolar H_2_O_2_ degraded per minute per 10^8^ CFU.

### Expression of catalase genes in *L. sakei* strains.

RNA isolation and cDNA synthesis were performed as described previously (28). A TRIzol Max bacterial RNA isolation kit (Invitrogen, CA, USA) was used according to the manufacturer’s instructions for RNA isolation. Sequences encoding the catalase of *L. sakei* ob4.1 were used as templates for primer design (Table S1). The relative expression of the *katA* gene was calculated using the comparative threshold (ΔΔ*C_T_*) method. *gapdh* was used as the reference gene.

### Caco-2 cell culture.

Caco-2 cells (ATCC, MD, USA) were grown on minimal essential medium (MEM) (Gibco, MA, USA) containing 10% fetal bovine serum, 1% GlutaMAX (Gibco, MA, USA), 1% MEM nonessential amino acids (Gibco, MA, USA), and 1% sodium pyruvate at 37°C and 5% CO_2_. After reaching confluence, the cells were seeded into 6- or 96-well plates at a density of 1 × 10^5^ cells/well for individual experiments and then cultured for 21 days (on average), with the medium changed every other day until the cells had differentiated.

### Preparation of bacterial suspension.

*Lactobacillus* strains were grown anaerobically in MRS medium for 48 h at 37°C, and then the bacterial cultures were pelleted down and resuspended in MEM (Gibco, MA, USA) to a final concentration of 10^8^ CFU/ml.

### Palmitate treatment.

Differentiated Caco-2 cells were treated with palmitate (2.5, 5, and 7.5 mM; Sigma-Aldrich) or fatty acid-free bovine serum albumin (Sigma-Aldrich, MO, USA) and incubated at 37°C and 5% CO_2_ for 6 h. Suspensions of *Lactobacillus* strains containing 10^8^ CFU/ml were added to each well and incubated for another 4 h. The Caco-2 cells were washed twice with PBS, and then the cell pellets were resuspended in 1 ml of TRIzol reagent (Molecular Research Centre, OH, USA) for subsequent RNA extraction.

### Adhesion assay in Caco-2 cells.

Caco-2 cells were subcultivated in 6-well plates as described above until confluence (3 to 5 days). The monolayers of cells were washed with Dulbecco’s PBS (DPBS), and new cell medium with or without 0.5% H_2_O_2_ was placed in each well. Then, bacterial suspensions were added to each well, with a final concentration of 10^6^ CFU in 1 ml of medium, and incubated at 37°C for 1 h. The cells were washed five times with DPBS to remove nonadherent bacteria. Cells were treated with trypsin-EDTA, and the number of viable bacterial cells was determined by the spread plate method on MRS medium. Then, the cells were incubated at 37°C for 48 h. The bacterial adhesion rate was calculated as number of adhered bacteria/total number of bacteria added × 100 (%).

### ROS measurement in Caco-2 cells.

Caco-2 cells were grown in 96-well plates as described above. ROS generation in the cells was measured using a cellular ROS assay kit (Abcam, Cambridge, UK) according to the manufacturer’s instructions.

### Mouse experiments.

The Institutional Animal Care and Use Committee at CHA University approved the animal experiments performed in this study. Specific-pathogen-free male C57BL/6J mice, aged 6 weeks, were fed either a 10% fat control diet (D12450B; Research Diets, Inc., NJ, USA) or a 60% fat treatment diet (D12492; Research Diets, Inc.) throughout the experiment. Six mice were used for each group. Eight weeks after starting the diet, 1 × 10^9^ CFU/100 μl of *L. sakei* ob4.1 or *L. sakei* DSM 20017 was administered once by oral gavage, and the colonization of bacteria in the mouse colon contents was determined by homogenizing those contents in 1 ml of sterile PBS, serially diluting the samples, and eventually plating them on MRS medium plates. To investigate the role of an ROS scavenger, mice were given *N*-acetylcysteine (NAC) (Sigma-Aldrich, MO, USA) at 100 mg/liter in their drinking water throughout the experiment. To calculate a competitive index for CFU of *L. sakei* ob4.1 versus *L. sakei* DSM 20017, two mice were cohoused for 8 weeks, fed a high-fat or low-fat diet, and given NAC in their drinking water or given regular water. Then, the two mice were separated after an inoculation of 1 × 10^9^ CFU/100 μl of *L. sakei* ob4.1 or *L. sakei* DSM 20017. After the number of bacteria in the mouse colon contents was determined, the competitive index was calculated as the ratio of the number of *L. sakei* ob4.1 over that of DSM 20017. Four pairs of mice (8 mice) were used for each experiment. To investigate the effects of long-term administration of the strains, the mice were orally treated with a 1 × 10^9^ CFU/100 μl mixture of the designated *L. sakei* strains or a mock suspension beginning 4 weeks after starting either diet and continuing every day for 4 weeks. The mice were subsequently euthanized, and samples were collected 24 h after the final infection.

### Bacterial culture.

Spontaneous mutants resistant to rifampin were isolated using the method of Chiaramonte et al. (53). Briefly, *L. sakei* strains were plated on MRS medium containing 100 mg/liter rifampin (Rif; Sigma-Aldrich, MO, USA) and incubated at 37°C for 4 to 5 days. For supplementation in mice, fresh cultures of *L. sakei* ob4.1-Rif and *L. sakei* DSM 20017-Rif were grown in MRS broth containing 100 mg/liter rifampin. The bacterial cells were pelleted and washed with PBS. The final concentration of each strain was 1 × 10^9^ CFU/ml.

### Measurement of colon tissue ROS.

The level of ROS in colon tissues was assessed in a piece of proximal colon tissue from the mice using an ROS fluorometric assay kit (Elabscience, TX, USA) according to the manufacturer’s instructions.

### Statistical analyses.

For clinical data, normally distributed continuous data are expressed as the mean ± standard deviation (SD), and nonnormally distributed data are expressed as the median and interquartile range. Categorical data are represented as the number and percent. A Kolmogorov-Smirnov goodness-of-fit test was performed to determine the population normality of each group. Characteristics between the healthy control group and obese group were compared using the Student’s *t* test or chi-square test. *Lactobacillus* CFUs between the two groups were compared using the Mann-Whitney test, and the prevalences of a positive culture for each strain were compared using Fisher’s exact test. The participants were categorized into four groups based on the interquartile ranges of their total fat intake, and fecal ROS levels were compared using one-way analysis of variance (ANOVA) followed by Bonferroni’s multiple-comparison test. Odds ratios (ORs) and 95% confidence intervals (CIs) for the prevalence of a positive culture of *L. sakei* species were calculated by a multivariate logistic regression analysis adjusted for age, sex, and BMI. A Pearson correlation analysis was performed to evaluate the relationship between fecal ROS levels and the relative abundance of *L. sakei* species. For the *in vitro* and mouse experiments, fold changes in ratios (mRNA levels and fecal ROS levels) and bacterial numbers were transformed logarithmically before analysis to normalize the data. One-way ANOVA followed by Tukey’s multiple-comparison tests were used to determine differences across the groups. Statistical analyses were performed using the Statistical Package for Social Science version 20.0 (SPSS Inc., IL, USA) and GraphPad Prism 8.0 (GraphPad Software, CA, USA). Statistical significance was defined as a *P* value of <0.05.

### Data availability.

16S rRNA gene amplicon sequencing data have been deposited with links to BioProject under accession number PRJNA541572 in the NCBI BioProject database. The complete genome sequence of *L. sakei* ob4.1 has been deposited in NCBI under the GenBank accession number CP075489.
