# Supplementary material for: High-Fat-Diet–Induced Oxidative Stress Linked to the Increased Colonization of Lactobacillus sakei in an Obese Population
Source: Microbiol Spectr. 2021 Jun 30;9(1):10.1128/spectrum.00074-21. doi: 10.1128/spectrum.00074-21 (PMC8552675; doi:10.1128/spectrum.00074-21)
Supplement: SUPPLEMENTAL FILE 1 — Download SPECTRUM00074-21_Supp_1_seq1.pdf, PDF file, 0.7 MB [file spectrum00074-21_supp_1_seq1.pdf]

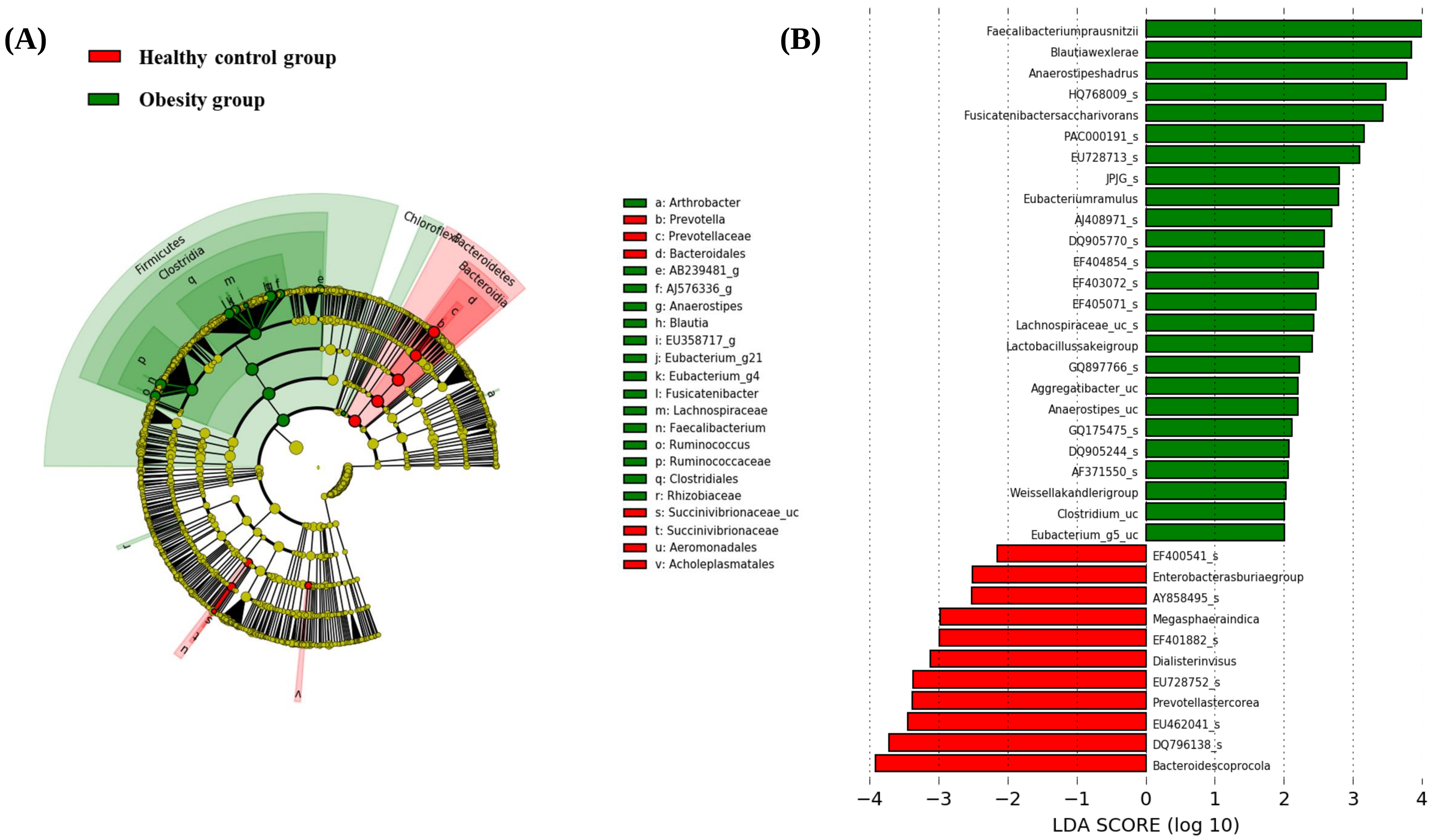
(A)
Healthy control group
Obesity group
Firmicutes
Clostridia
Chloroflexi
Bacteroidetes
Bacteroidia
a: Arthrobacter
b: Prevotella
c: Prevotellaceae
d: Bacteroidales
e: AB239481_g
f: AJ576336_g
g: Anaerostipes
h: Blautia
i: EU358717_g
j: Eubacterium_g21
k: Eubacterium_g4
l: Fusicatenibacter
m: Lachnospiraceae
n: Faecalibacterium
o: Ruminococcus
p: Ruminococcaceae
q: Clostridiales
r: Rhizobiaceae
s: Succinivibrionaceae_uc
t: Succinivibrionaceae
u: Aeromonadales
v: Acholeplasmatales
(B)
Faecalibacteriumprausnitzii
Blautiawexlerae
Anaerostipeshadrus
HQ768009_s
Fusicatenibactersaccharivorans
PAC000191_s
EU728713_s
JPJG_s
Eubacteriumramulus
AJ408971_s
DQ905770_s
EF404854_s
EF403072_s
EF405071_s
Lachnospiraceae_uc_s
Lactobacillussakeigroup
GQ897766_s
Aggregatibacter_uc
Anaerostipes_uc
GQ175475_s
DQ905244_s
AF371550_s
Weissellakandlerigroup
Clostridium_uc
Eubacterium_g5_uc
EF400541_s
Enterobacterasburiaegroup
AY858495_s
Megasphaeraindica
EF401882_s
Dialisterinvisus
EU728752_s
Prevotellastercorea
EU462041_s
DQ796138_s
Bacteroidescoprocola
−4 −3 −2 −1 0 1 2 3 4
LDA SCORE (log 10)

Figure S1.

```
98.5% identity in 479 residues overlap; Score: 2522.0; Gap frequency: 0.0%

DSM20017_K      1 MTNQLTTNEGQPWADNQHSQTAGQRGPVLIQDYQLLEKLAHFNRERIPERVVHAKGAGAK
Ob4.1_KatA      1 MTNQLTTNEGQPWADNQHSQTAGQRGPVLIQDYQLLEKLAHFNRERIPERVVHAKGAGAK
                  ************************************************************

DSM20017_K     61 GYFKVTKDMSAYTKAAVFSGVGKKTPLITRFSQVAGEAGYPDTYRDVRGFAVKFYTEEGN
Ob4.1_KatA     61 GYFKVTKDMSAYTKAAVFSGVGKKTPLITRFSQVAGEAGYPDTYRDVRGFAVKFYTEEGN
                  ************************************************************

DSM20017_K    121 YDIVGNNTPVFFVNDPLKFPDFIHSQKRDPRTHARSQDMQWDFWSLSPESVHQVTILMSD
Ob4.1_KatA    121 YDIVGNNTPVFFVNDPLKFPDFIHSQKRDPRTHARSQDMQWDFWSLSPESVHQVTILMSD
                  ************************************************************

DSM20017_K    181 RGIPASYRMMHGFGSHTFKWVNAQGEQFWVKYHFKTNQGIHNLSNELADELAGKDTDYLQ
Ob4.1_KatA    181 RGIPTSYRMMHGFGSHTFKWVNAQGEQFWVKYHFKTNQGVHNLSNELADELAGKDTDYLQ
                  **** ********************************** ********************

DSM20017_K    241 NDLFDAIETGDYPSWTVAVQLVPYEDGLNYPQDIFDVTKVISQKDYPLIEIGQMVLDENP
Ob4.1_KatA    241 NDLFDAIETGDYPSWTVAVQLVPYEDGLNYPQDIFDVTKVISQKDYPLIEIGQMVLDENP
                  ************************************************************

DSM20017_K    301 TNNFEDIEELAFSPANLVPGIEASPDKLLQGRLFGYKDAERYRLGANYEQLPVNRPKVPV
Ob4.1_KatA    301 TNNFEDIEELAFSPANLVPGIEASPDKLLQGRLFGYKDAERYRLGANYEQLPINRPKVPV
                  **************************************************** *******

DSM20017_K    361 HNYERDGAMAQNQATGVNYEPNSQDGPTEVPAAKIHGDQLSGTTGNFSADPDYYSAAGKL
Ob4.1_KatA    361 HNYERDGVMAQNQETGVNYEPNSQDGPTEVPAAKIHSDQLSGTTGNFSTDPDYYSAAGKL
                  ******* ***** ********************** *********** ***********

DSM20017_K    421 YRLLSADEQTRLIENIRMNLGQVTKPEIQIREVKQFYQADPEYGRRVATALNLDLAQFE
Ob4.1_KatA    421 YRLLSADEQTRLIENIRMNLGQVTKPEIQIREVKQFYQADPEYGRRVATALNLDLAQFE
                  ***********************************************************
```

Figure S2.

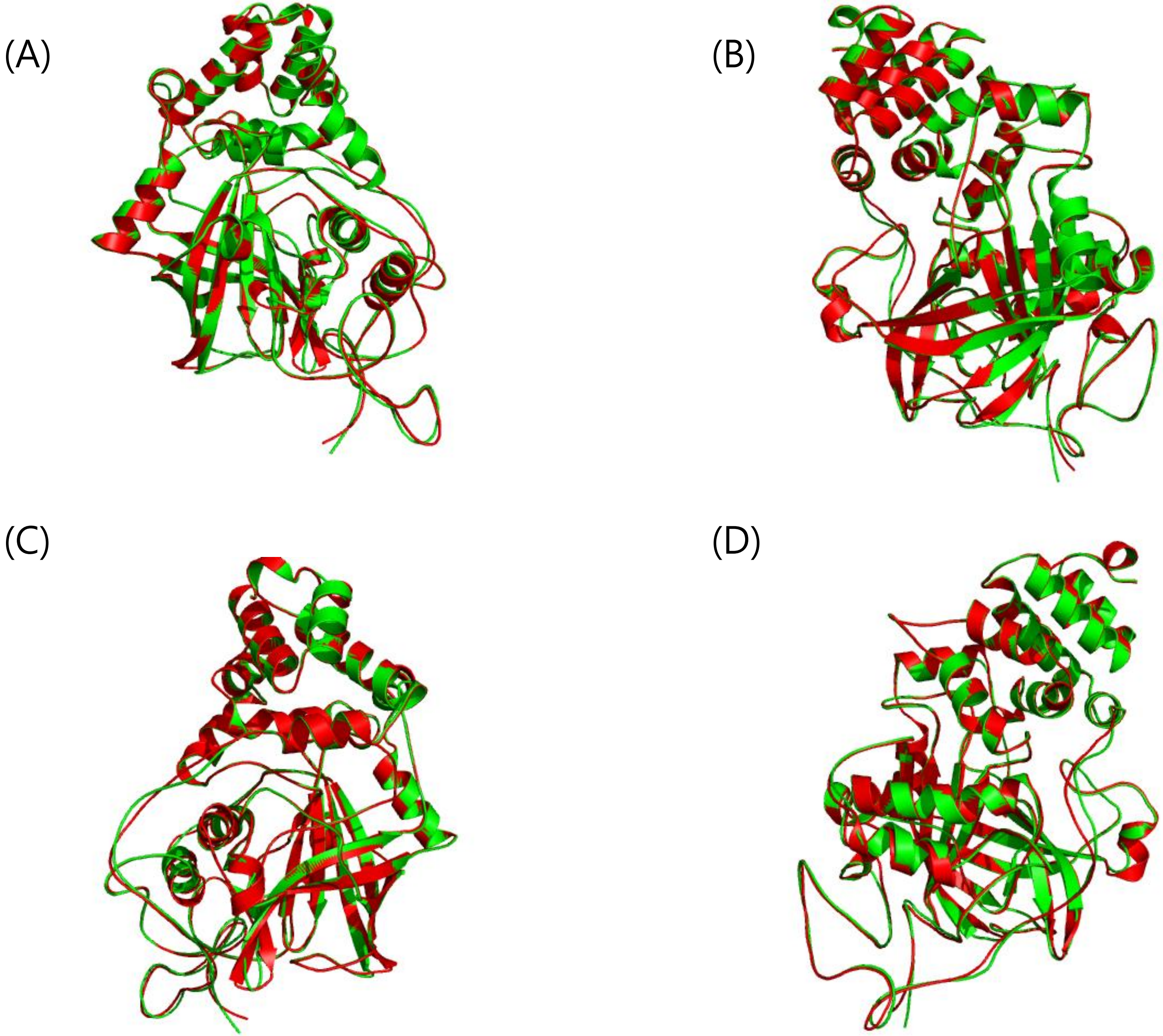
(A)
(B)
(C)
(D)

Supplementary Figure legends

Figure S1. Comparison of the fecal microbiota profile of healthy and obese subjects

Microbiota profiling was performed using DNA isolated from the feces of subjects, and differences in taxa composition were compared using the LDA effect size algorithm. Green, elevated in obese subjects compared with healthy subjects; red, reduced in obese subjects compared with healthy subjects. (A) The cladogram shows differences in taxa composition determined by microbiota profiling of feces from the healthy control group and obese group. (B) Species with significant differences that have an LDA score greater than the estimated value; the default score is 2.0.

Figure S2. Amino acid sequence alignment of KatA genes in *L. sakei* ob4.1 and *L. sakei* DSM 20017

KatA genes expressed in *L. sakei* ob4.1 and DSM 20017 were 98.5% identical based on amino-acid sequences.

Figure S3. Superposition of predictive structure models of catalase enzymes of *L. sakei* ob4.1 (Green) and DSM 20017 (Right) based on the amino sequences.

Four different views of the superimposed structures are given with rotations of 0° (A), 90° (B), 180° (C) and 270° (D):

Table S1. List of primers used in the study

Organism	Taret gene	Sequence (5'-3')
Genus Lactobacillus	16s rRNA	F:AGCAGTAGGGAATCTTCCA
		R: CACCGCTACACATGGAG
L.sakei	16s-23s rRNA	F: GAGCTAATCCCCCATAATGAAACTAT
		R: GATAAGCGTGAGGTCGATGGTT
	katA	F: AATTGCCTTCTTCCGTGTA,
		R: AGTTGCGCACAATTATTTTC
L.paracasei	16s-23s rRNA	F: ACATCAGTGTATTGCTTGTCAGTGAATAC
		R: CCTGCGGGTACTGAGATGTTTC
L.casei	16s-23s rRNA	F: CTTCCTGCGGGTACTGAGATGT
		R: CTATAAGTAAGCTTTGATCCGGAGATTT
L.rhamnosus	16s-23s rRNA	F: CGGCTGGATCACCTCCTTT
		R: GCTTGAGGGTAATCCCCTCAA
L.gasseri	16s-23s rRNA	F: AGCGACCGAGAAGAGAGAGA
		R: TGCTATCGCTTCAAGTGCTT
L.salivarius	16s-23s rRNA	F: GTCGTAACAAGGTAGCCGTAGGA
		R: TAAACAAAGTATTCGATAAATGTACAGGTT
L.plantarum	16s-23s rRNA	F: TGGATCACCTCCTTTCTAAGGAAT
		R: TGTTCTCGGTTTCATTATGAAAAAATA
L.reuteri	16s-23s rRNA	F: ACCGAGAACACCGCGTTATTT
		R: CATAACTTAACCTAAACAATCAAAGATTGT

		CT
L.fermentum	16s-23s rRNA	F: ACTTAACCTTACTGATCGTAGATCAGTCA
		R: AACCGAGAACACCGCGTTAT
L.brevis	16s-23s rRNA	F: ATTTTGTTTGAAAGGTGGCTTCGG
		R: ACCCTTGAACAGTTACTCTCAAAGG
Bacteria	16s	27F: AGAGTTTGATCMTGGCTCAG
		1492R: TACGGYTACCTTGTTACGACTT
	V3-V4 region	341F:TCGTCGGCAGCGTC AGATGTGTATAAGAGACAG-CCTACGGGNGGCWGCAG
		805R:GTCTCGTGGGCTCGGAGATGTGTATAAGAGACAG-GACTACHVGGGTATCTAATCC

Table S2. The list of reference strains used in this study

Species	Reference strains
L. sakei	DSM 20017
L. paracasei	DSM 20020
L. casei	NCDO161
L. rhamnosus	ATCC 53103
L. gasseri	DSM 20243
L. salivarius	DSM 20554
L. plantarum	DSM 10667
L. reuteri	DSM 20016
L. fermentum	ATCC 14931
L. brevis	DSM 20054

Table S3. Basic characteristics of the subjects

Characteristics	Control subjects (n=64)	Obesity (n=88)	P value
Age (year)	38.28±10.38	40±9.43	0.22
Gender [n (%)]			0.20
Male	31 (48.4)	51 (58.0)	
Female	33 (51.6)	37 (42.0)	
Regular exercise [n (%)]	28 (43.8)	33 (37.5)	0.24
Regular alcohol Drinking [n (%)]	21 (32.8)	38 (43.2)	0.13
Current smoking [n (%)]	0 (0)	0 (0)	NS
BMI (kg/m^2)	20.20±3.16	28.23±1.14	<0.01
Body fat (%)	24.57±6.11	34.49±4.13	0.02
Total calorie intake (kcal/day)	2354.78±118.43	3457.20±315.58	0.01
Carbohydrate (g/1000kcal/day)	162.23±3.15	205.34±9.78	0.08
Fiber (g/1000kcal/day)	14.81±0.67	15.55±0.89	0.23
Protein (g/1000kcal/day)	41.21±3.15	46.22±3.57	0.34
Fat (g/1000kcal/day)	23.55±3.35	41.25±5.98	<0.01
Saturated fat (g/1000kcal/day)	7.34±0.99	19.28±5.01	<0.01
Mono-unsaturated fat (g/1000 kcal/day)	7.53±1.12	13.57±0.54	0.07
Poly-unsaturated fat (g/1000kcal/day)	5.78±1.24	6.38±0.98	0.64

Data are shown as the mean ± standard deviation or the number (percentage).

Abbreviation: BMI, Body mass index; NS, no statistically significant difference.

Regular exercise was defined as activity more than a moderate degree (walking more than 5 times/week for 30 min, moderate-intensity physical activity more than 5 times/week for 30 min, or vigorous-intensity physical activity more than 3 times/week for 20 min) in recent 1 week. Smoking was defined as being a current smoker and alcohol drinking was defined as drinking alcoholic beverage ≥ 70 g/day, or more frequent than once a week. Energy adjustment was performed by calculating nutrient density and expressed as intake / 1000kcal.

P values are calculated by using Student's *t* test or chi square test.

Table S4. The prevalence of *Lactobacillus* species in faeces of healthy control and obesity group

Lactobacillus species	Control subjects (n=64)	Obesity (n=88)	P value
L. sakei	7 (10.9)	30 (34.1)	<0.01
L. paracasei	10 (15,6)	11 (12.5)	0.31
L. casei	9 (14.1)	15 (17.0)	0.54
L. rhamnosus	8 (12.5)	8 (9.1)	0.31
L. gasseri	7 (10.9)	13 (14.8)	0.15
L. salivarius	5 (7.8)	2 (2.3)	0.48
L. plantarum	5 (7.8)	8 (9.1)	0.75
L. reuteri	4 (6.3)	10 (11.4)	00.19
L. fermentum	2 (3.1)	5 (5.7)	0.28
L. brevis	2 (3.1)	3 (3.4)	0.43

Data are shown as the number (%). The prevalence of Lactobacillus species was determined using species-specific PCR. P values were calculated by Fisher’s exact test.

Table S5. Clinical characteristics of the host subjects of L.sakei ob4.1

Variables	Values
Age (years)	35
Gender	Male
BMI (kg/m^2)	32.15
Regular exercise	Yes
Regular alcohol drink	No
Calorie intake (kcal/day)	3035 kcal/day
Carbohydrate intake (g/1000kcal/day)	194.21
Protein intake (g/1000kcal/day)	43.54
Fiber intake (g/1000kcal/day)	15.67
Fat intake (g/1000kcal/day)	40.28
Saturated fat intake (g/1000kcal/day)	18.46
Mono-unsaturated fat (g/1000kcal/day)	11.13
Poly-unsaturated fat (g/1000kcal/day)	6.18
